# The Infection Timeline in Solid Organ Transplantation: Lessons from the Swiss Transplant Cohort

**DOI:** 10.1007/s11908-026-00885-3

**Published:** 2026-06-04

**Authors:** Christian van Delden, Ming Zou, Nicolas J Mueller

**Affiliations:** 1 Transplant Infectious Diseases Unit, Service of Infectious Diseases, University Hospitals Geneva, University of Geneva, Geneva, Switzerland; 2 Swiss Transplant Cohort Study, University Hospital Basel, Basel, Switzerland; 3 Present Address: Department of Infectious Diseases and Hospital Epidemiology, University Hospital Zurich, University of Zurich, Zurich, Switzerland

**Keywords:** Swiss Transplant Cohort Study, Epidemiology, Transplant Infectious Diseases, Timeline

## Abstract

**Purpose of Review:**

Understanding the timeline of infections after solid organ transplantation is essential for prevention, diagnosis, and appropriate empiric therapy. Preventive strategies have substantially reduced some infections, while the increasing prevalence of multidrug-resistant organisms challenges traditional empiric approaches.

**Recent Findings:**

Contemporary epidemiology demonstrates a shift in post-transplant infections. Opportunistic infections have become uncommon, while bacterial infections now predominate, particularly those caused by Enterobacterales, non-fermenting gram-negative bacilli, and gram-positive pathogens. The success in the control of opportunistic infections is increasingly challenged by the rise of multidrug-resistant organisms.

**Summary:**

Strengthened strategies to prevent bacterial infections, especially those due to multidrug-resistant pathogens, are needed in solid organ transplant recipients.

## Introduction

Since the seminal work of the pioneers of transplant infectious diseases in 1998, the timeline of infections observed in solid organ transplantation recipients (SOTr) has informed the caregivers in their daily routine and was an indispensable tool helping to promptly diagnose and treat [1]. It importantly also influenced prevention strategies, which have been expanded and refined over the decades. These interventions, coupled with a more personalized approach to immunosuppression as well as improved diagnostics, had a profound impact on this timeline. This impact was already incorporated in the 2007 review by J. Fishman, where for the most intense phase (usually designated as month 1 to month 6 after transplantation), the characteristic infections were grouped according to type (antiviral, antibacterial, antiparasitic) of prevention applied [2]. Additional factors to weigh in are the course after transplantation (optimal vs. eventful clinical course, i.e. rejection) and early donor-derived infections. Rejection episodes interfere with the planned tapering of immunosuppression and maintain the recipient in a high-intensity immunosuppression phase. Knowledge of the timeline was particularly crucial as many of the observed infectious events were caused by opportunistic pathogens, which were less well known and not actively investigated in routine care [3].

In our personal experience, opportunistic infections have become less prevalent in recent years. On another note, the increase in multidrug resistance pathogens (primarily Enterobacterales and non-fermenters) has added an additional challenge to the care of these vulnerable patients. Together with more refined immunosuppressive protocols and stringent implementation of prevention, the hypothesis was that the timeline of infection further evolved over time.

Based on data from the longitudinal Swiss Transplant Cohort Study (www.stcs.ch), which enrolls all recipients of a solid organ in Switzerland since 2008, and collects relevant outcomes after transplantation, this review will try to shed light on a contemporary and real-life picture of the infectious burden after transplantation. Consequences for diagnostic and prevention strategies will be discussed.

## Burden and Timeline of Infections During the First Year

The first published comprehensive timeline did separate three relevant time periods: An early phase (first month) with conventional nosocomial infections, a phase with unconventional or opportunistic infections (month 2 to moth 6) and a phase starting 6 months post-transplant with community-acquired and some reactivations of latent persistent infections [1]. The emphasis was on opportunistic infections, with prevention concepts just being developed. Their widespread implementation led to a shift in the observed infections, as both trimethoprim-sulfamethoxazole (TMP-SMX) and (val)-ganciclovir (VAL) have (desired) collateral effects in addition to their main roles. Besides *Pneumocystis jirovecii* and toxoplasmosis, TMP-SMX targets *Listeria monocytogenes*, some Nocardia strains, and other bacterial pathogens. (Val)-ganciclovir protects against additional herpes viruses such as Herpes simplex virus and Varicella zoster virus [4]. The new timeline proposed in 2007 reflected the role of prevention. In addition, the importance of the clinical course after transplantation in the trajectory of the patient was emphasized [2].

Most of the data available on the frequency of infectious diseases (ID) events following transplantation is fragmented, focuses on specific infections, and is restricted to a specific organ. The open-ended longitudinal Swiss Transplant Cohort Study with a prospective systematic collection of ID events, based on pre-specified definitions, constituted a unique opportunity to have real-life insights into the burden of infectious diseases during the first year post-transplant in the current era [5]. The first patient was enrolled in May 2008, and since, > 92% of all patients transplanted in Switzerland consented to systematic data collection after transplantation.

**Fig. 1:**
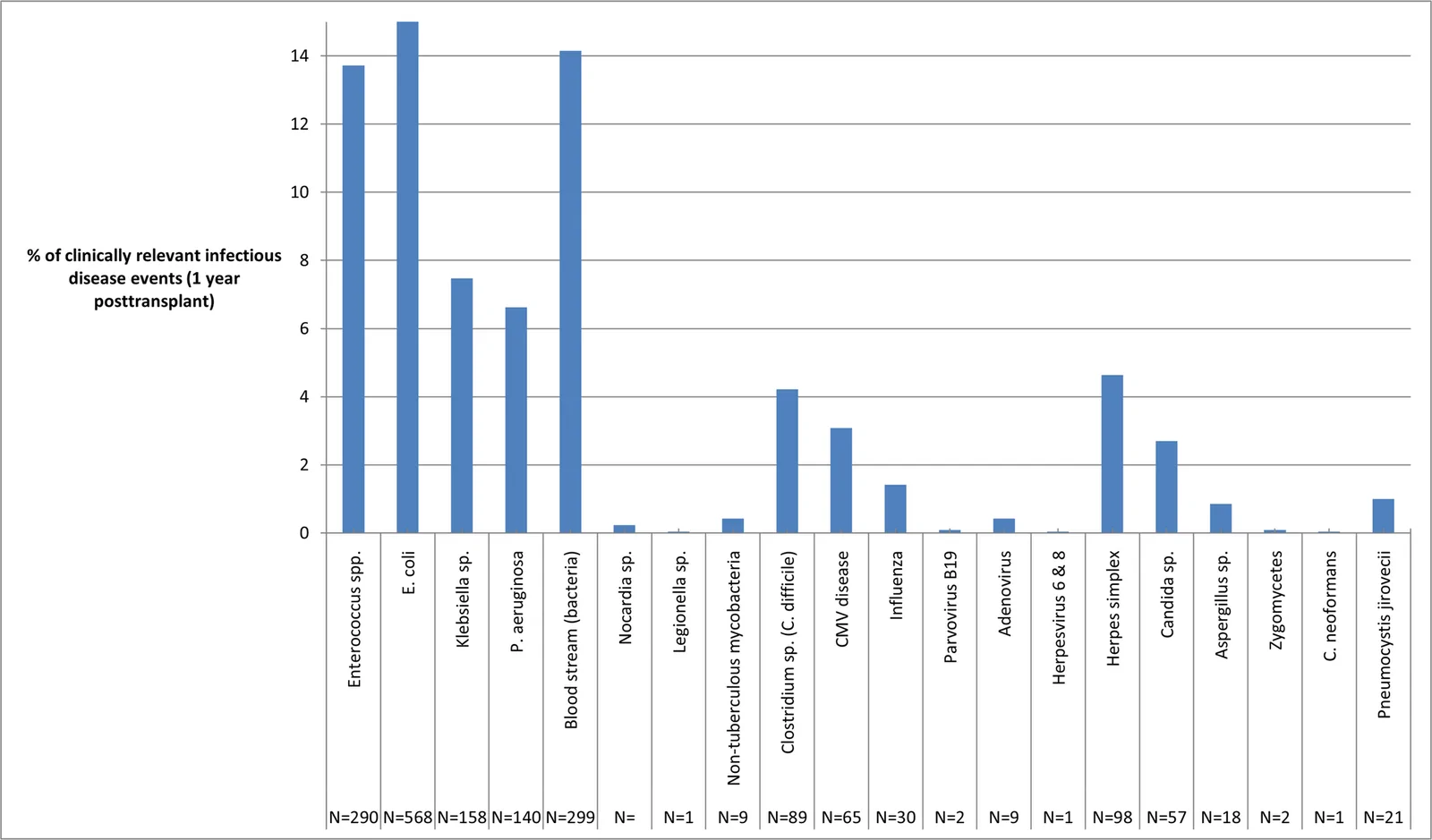
Percentage of infectious diseases events by selected pathogens or sites in relation to the overall number

**Fig. 2:**
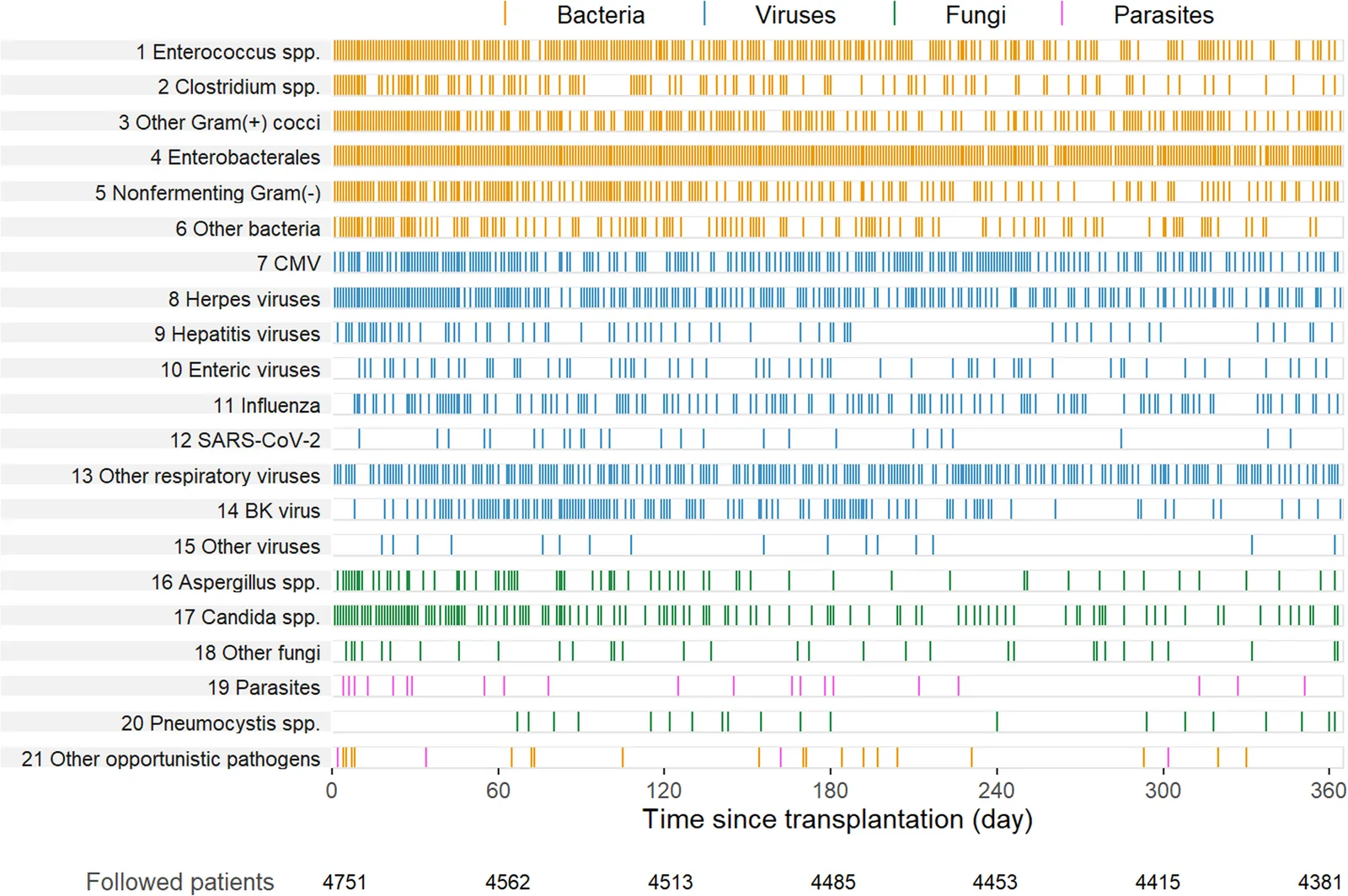
Timeline of selected infectious diseases events in the first year after transplantation. A total of 5389 clinicallyrelevant infections were recorded in 4751 SOTr (383 heart-, 1121 liver-, 488 lung-, 2646 kidney- and 113 kidney-pancreas SOTr)

**Fig. 3:**
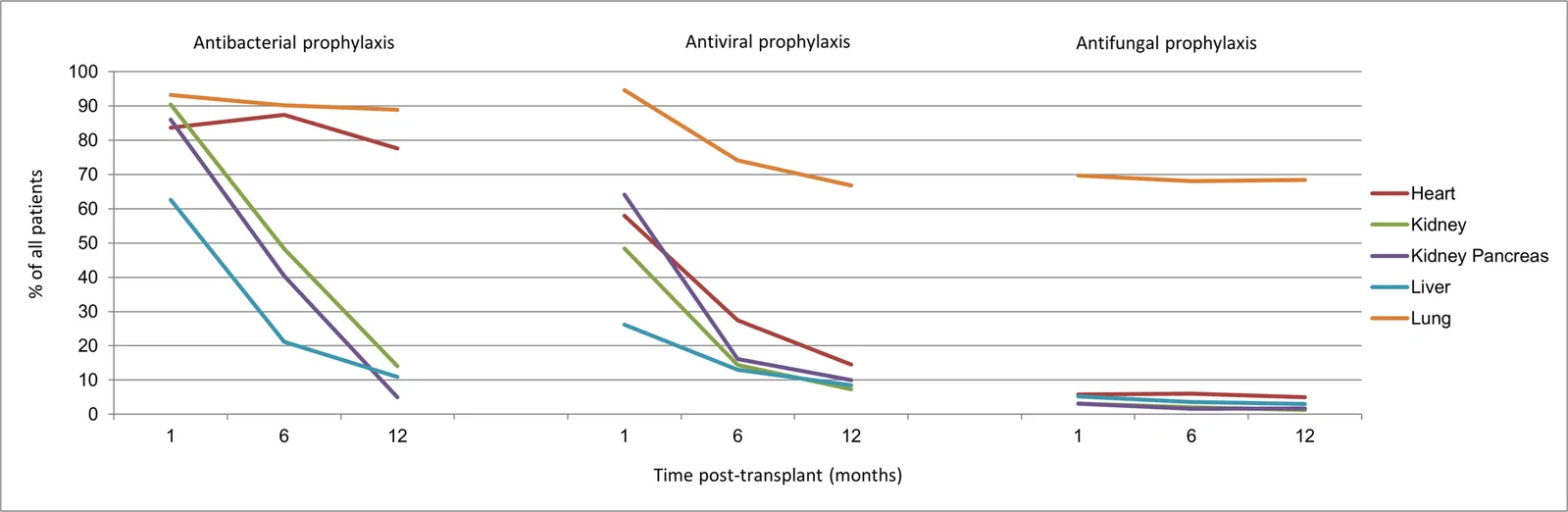
Percentage of transplant recipients receiving antibacterial, antiviral, or antifungal prophylaxis at 1, 6, and 12months after transplantation, stratified by transplanted organ

The first burden of infection study included 2761 SOTr with up to one year of follow-up. A total of 3520 clinically-relevant infectious diseases events, all occurring during the first year, were analyzed [6]. Among the enrolled participants, 60% were kidney, 21% liver, 2% pancreas, 10% lung, and 7% heart transplant recipients. The main result of the study is exemplarily shown in Fig. 1, where the percentage of ID events in relation to the overall number are shown. The low relative number of infections by opportunistic pathogens contrasted with a higher number of infections with common bacterial pathogens. An updated analysis is shown in Fig. 2, where the 5389 clinically relevant infections were recorded in 4751 SOTr in the first 12 months. Figure 3 shows antibacterial, antiviral and antifungal prophylaxis administered over time in the different organ recipients in the first year after transplantation. With the exception of lung recipients, where a majority of patients still received antifungal and antiviral prophylaxis at one year, most recipients were without prophylaxis at one year.

### The main Findings can be summarized as follows

(1) Globally the burden of infections remains high during the entire first year with more than 50% of patients being infected. During the first month, clinically relevant infections occurred in 27% of SOTr (11.95 episodes per 1000 transplant-days), between 1 and 6 months 33% patients (3.88 episodes per 1000 transplant-days) and between 6 and 12 months posttransplant 23% patients (2.02 episodes per 1000 transplant-days) were infected.
(2) The prevention strategies work and opportunistic infections have become rare. With some exceptions opportunistic pathogens have become less prevalent during the most intense immunosuppression period. They now account for 2–8% of all infections. New emerging opportunistic pathogens include BK polyomavirus. Some opportunistic infections occur outside of their predicted time window (1–6 months post-transplant), once their prophylaxis is stopped as for example late onset cytomegalovirus (CMV) disease. Some pneumocystis infections occur at later time points, once prophylaxis has been discontinued, following treatment of acute rejections with increased immunosuppression [7]. Herpes virus infections are quite prominent in the absence of a specific prophylaxis as for instance in the setting of a preemptive surveillance for CMV infections [4].
(3) Being responsible for almost 60% of all infections, bacteria are the most prevalent pathogens and this remains so over the course of the first year. Sites and species vary depending on the transplanted organ, but the overall bacterial burden is very similar between the programs.
(4) The pathogens encountered are those primarily expected in a nosocomial setting. Thus, Enterobacterales and gram-positive bacteria predominate. This has implications for treatment, as multidrug resistance is more prevalent in these pathogens as compared to community acquired or opportunistic ones.

## Implications for Clinical Practice

These findings have implications for diagnosis, treatment and prevention. While in the initial differential diagnosis of a post-transplant infection, opportunistic pathogens still must be considered, they might not top the list in particular if a chemoprophylaxis is in place and correctly taken. Empiric therapy should take into account the local resistance pattern of the traditional bacterial pathogens, more likely to be a MDR bacteria, than an opportunistic pathogen. Preventive measures must be reinforced also for more “traditional” infections.

The previous high burden of opportunistic infections led to increasing awareness and the development of a specialized field focused on improving diagnosis and treatment [8]. These efforts had a great impact on morbidity and mortality after solid organ transplantation, with survival rates ever increasing. This success must now be preserved by a renewed attention on the prevailing role of more conventional infections.

The importance of preventing donor-transmitted infections has been underlined by reports from the STCS concerning fulminant herpes simplex virus (HSV) hepatitis in serologically negative liver recipients [9]. Such reports have led to modifications of the Swiss national guidelines now including systematic anti-HSV prophylaxis for serologically negative recipients transplanted from serologically positive donors (R-/D+). Since 2012, any infectious disease episode can be labelled as potentially donor-derived infection. A project embedded in the STCS is currently analyzing in detail the approximately 250 episodes with a potential donor-derived etiology and carefully reviewing all patient data to categorize all episodes and analyze the impact on patient and graft survival.

The concept of “indirect effects” has been developed to describe untoward effects not directly linked to an infectious process (such as tissue damage due to direct infection), but to downstream effects believed to be the result of a changed immunological milieu. Cytomegalovirus is probably the most robust example, with effects on the graft (rejection) or occurrence of other infection (invasive fungal infections) seen following the initial hit [10]. Recent research has shown similar effects also for more traditional infections. In a study on the incidence and risk factors for *Clostridioides difficile* infection after SOT, such an episode was associated with a higher likelihood of a (delayed) graft loss [11].

Surgical site infections are an important but somewhat neglected complication after solid organ transplantation. In a recent study also originating from the STCS, surgical site infections after liver transplantation had a relevant impact on graft survival [12]. The role of the preservation fluid is much discussed, as current practices regarding culturing vary widely between countries. It is planned to perform a cluster-randomized study within the STCS and international centers to answer this question. It highlights an additional benefit of such a collaborative effort: namely its potential to use it for interventional studies.

The burden of multidrug-resistant (MDR) bacteria has been, so far, low in Swiss SOT recipients. An analysis of bacterial isolates between 2008 and 2014 showed a rate of 15% for extended-spectrum beta-lactamase (ESBL) producing Enterobacterales These numbers were stable up to 2019, with 13% of Enterobacterales being ESBL producers [13]. The occurrence of carbapenemase-producing Enterobacterales (CPE) was rare. This is of course not the case for other geographic eras. Several studies have addressed the role of MDR in solid organ transplantation. One paper assessed the impact on infectious episodes, mortality and graft loss of MDR-colonized solid organ transplant recipients in comparison to non-colonized recipients. In this systematic review and meta-analysis, 39 studies with over 15’000 recipients were analyzed [14]. Compared to non-colonized recipients, colonization with multidrug resistant bacteria was associated with a higher risk of infections and mortality. A further study reported that in hospitalized patients with a carbapenem-resistant Enterobacterales isolate grouped according to whether they were a recipient of a solid graft or not, receipt of an organ was not associated with a higher mortality regardless if the isolate was considered an infection or colonization [15]. Mortality, however, was high in both (transplant and non-transplant) groups. Essentially, both large studies come to a similar conclusion: MDR is increasing your risk for an unfavorable outcome. One avenue increasingly chosen is to actively search for colonization with MDR pathogens before transplantation, in both donor and recipient. A recent study has shown that with such an approach, even very difficult to treat organisms can be anticipated and measures taken, resulting in good outcomes [16].

### How can Prevention be Improved? Multiple Pillars need to be Addressed

The specific prevention strategies aimed at opportunistic infections have been mentioned. A rigorous adherence to the protocols is essential.

For the prevention of nosocomial infections, the strategies applicable are not different from those used for all patients. It is essential to raise awareness of its significance, as it has direct and potentially life-critical implications in the context of transplantation. Following a short (24–72 h) peri-transplant antibacterial prophylaxis, specifically directed towards the local recipient flora, and potentially also adapted to donor colonization by MDR isolates, adequate source control including drainage of all accessible sites remains essential. Specific risk factors for early post-operative infections have been described and should guide patient tailored prevention strategies [17].

An optimal preparation of the candidate is essential. A STCS study concluded that one in ten SOT recipients experience at least one episode of a vaccine-preventable infection (VPI) post-transplantation [18]. The occurrence of a VPI was associated with an increased risk of death and/or graft-loss, highlighting the potential devastating effect. When the relative risk of occurrence of a VPI in SOT recipients was compared to the general population, using age-adjusted standardized incidence ratios, the standardized incidence ratio for notifiable VPIs in the SOT population was more than 27 times higher than in the general population.

## Burden and Timeline of Infections After the First Year

So far data on infections after the first-year post-transplant are scarce; limited to single center or single transplant type experiences and short observation periods (402 days to 4 years post-transplant). Globally the incidence of infections has been reported around 0.4 infections per 1000 transplant days [19–21]. However, these studies do not provide a global picture of the burden and timeline of clinically relevant infections over all transplant programs over a long post-transplant observation period. Obviously, the adequate capture of late complications years after transplant is rendered difficult by the outpatient care of SOTr over time and potential treatments of infections outside of transplant reference centers. With over 9’000 SOTr included and a median follow-up time exceeding 5 years, the STCS with its unique longitudinal long-term design and efficient capture of all clinically relevant infections due to the particularly dense Swiss medical network, will be the ideal tool to provide such data.

## Summary

The timeline of infections after solid organ transplantation has undergone constant changes since it was first described. Opportunistic infections were successfully reduced by wide implementation of prevention strategies. The current main burden are bacterial infections caused by traditional pathogens found in nosocomial infections. Sites and species vary according to transplanted organ. Mid- and long-term effects on graft and patient survival can be demonstrated. Efforts to reduce these infections must be increased, and awareness risen for these potentially avoidable complications.

## Key References

- Fishman, J.A. and R.H. Rubin, Infection in organ-transplant recipients. N Engl J Med, 1998;338(24):1741-51.
  - Seminal paper introducing the conept of a timeline of infections after solid organ transplantation.
- Fishman, J.A., Infection in solid-organ transplant recipients. N Engl J Med, 2007;357(25):2601-14.
  - Evolution of the timeline, taking into account preventive measures, and the clinical course after transplantation.
- Van Delden, C., et al., Burden and Timeline of Infectious Diseases in the First Year After Solid Organ Transplantation in the Swiss Transplant Cohort Study. Clin Infect Dis, 2020;71(7):e159-e169
  - Real-life map of infections after solid organ transplantation in the first year, in a nationwide transplant cohort study.

## Data Availability

No datasets were generated or analysed during the current study.
